# MASiVEdb: the Sirevirus Plant Retrotransposon Database

**DOI:** 10.1186/1471-2164-13-158

**Published:** 2012-04-30

**Authors:** Alexandros Bousios, Evangelia Minga, Nikoleta Kalitsou, Maria Pantermali, Aphrodite Tsaballa, Nikos Darzentas

**Affiliations:** 1 Institute of Agrobiotechnology, Centre for Research and Technology Hellas, Thessaloniki, 57001, Greece; 2 Department of Genetics and Plant Breeding, Aristotle University of Thessaloniki, Thessaloniki, 54006, Greece; 3 Central European Institute of Technology, Masaryk University, Brno, Czech Republic

## Abstract

**Background:**

Sireviruses are an ancient genus of the *Copia* superfamily of LTR retrotransposons, and the only one that has exclusively proliferated within plant genomes. Based on experimental data and phylogenetic analyses, Sireviruses have successfully infiltrated many branches of the plant kingdom, extensively colonizing the genomes of grass species. Notably, it was recently shown that they have been a major force in the make-up and evolution of the maize genome, where they currently occupy ~21% of the nuclear content and ~90% of the *Copia* population. It is highly likely, therefore, that their life dynamics have been fundamental in the genome composition and organization of a plethora of plant hosts. To assist studies into their impact on plant genome evolution and also facilitate accurate identification and annotation of transposable elements in sequencing projects, we developed MASiVEdb (Mapping and Analysis of SireVirus Elements Database), a collective and systematic resource of Sireviruses in plants.

**Description:**

Taking advantage of the increasing availability of plant genomic sequences, and using an updated version of MASiVE, an algorithm specifically designed to identify Sireviruses based on their highly conserved genome structure, we populated MASiVEdb (http://bat.infspire.org/databases/masivedb/) with data on 16,243 intact Sireviruses (total length >158Mb) discovered in 11 fully-sequenced plant genomes. MASiVEdb is unlike any other transposable element database, providing a multitude of highly curated and detailed information on a specific genus across its hosts, such as complete set of coordinates, insertion age, and an analytical breakdown of the structure and gene complement of each element. All data are readily available through basic and advanced query interfaces, batch retrieval, and downloadable files. A purpose-built system is also offered for detecting and visualizing similarity between user sequences and Sireviruses, as well as for coding domain discovery and phylogenetic analysis.

**Conclusion:**

MASiVEdb is currently the most comprehensive directory of Sireviruses, and as such complements other efforts in cataloguing plant transposable elements and elucidating their role in host genome evolution. Such insights will gradually deepen, as we plan to further improve MASiVEdb by phylogenetically mapping Sireviruses into families, by including data on fragments and solo LTRs, and by incorporating elements from newly-released genomes.

## Background

The intense activity of long terminal repeat (LTR) retrotransposons has been among the major drivers (together with polyploidization events) for the often enormous size of plant genomes [1-3]. This phenomenon was initially suggested to lead plants to ‘genomic obesity’ [4], before it was shown that mechanisms of LTR retrotransposon removal counterbalance this propensity [5-7]. The relative success of these two opposing forces likely underlies the impressive variation in the LTR retrotransposon content of plant genomes. Just over 7% of the tiny genome (125 Mb) of *Arabidopsis thaliana* is occupied by this transposable element (TE) type [8,9], in contrast to approximately 25% of the rice genome (389 Mb) [10], ~75% of the maize genome (2,300 Mb) [11], and ~65% of the wheat genome (16,000 Mb) [12]. Although these vast genomic stretches were long dismissed as ‘selfish’ or ‘junk’ DNA [13,14], they have eventually emerged as a major evolutionary force with profound effects not only on the structure, organization and composition of the host epi/genome, but also on the evolution, function, and regulation of genes [15-18].

Sireviruses are an ancient LTR retrotransposon genus of the *Copia* superfamily, and the only one (of either *Copia* or *Gypsy*) that has exclusively proliferated within the plant kingdom [19]. Due to their host specificity they were originally termed Agroviruses [19], before being renamed to Sireviruses (derived from the SIRE1 element of soybean [20]) by the International Committee on the Taxonomy of Viruses (ICTV) [21]. In contrast to ICTV which has divided the *Copia* (or *Pseudoviridae*) superfamily into three genera (i.e. Sireviruses, Hemiviruses and Pseudoviruses), the unified classification system for eukaryotic TEs [22] is devoid of an analogous genus-level taxonomy. This has left as yet open questions on the position of Sireviruses within the LTR retrotransposon order, and whether they should actually be considered as viruses. Based on all published work on Sireviruses so far [23-28], it is safe to assume that the characteristics of their life cycle correspond to that of typical LTR retrotransposons and not of viruses.

Sireviruses have infiltrated many phylogenetic branches of flowering plants, as several elements from a plethora of monocot and eudicot species have been classified as Sireviruses [23,27] (Additional file 1: Figure S1). More specifically, and based on the Sirevirus origin [23] of abundant families in rice [29], barley and wheat [12,30], they have extensively colonized the genomes of grasses. Notably, Sireviruses currently take up ~21% of the maize genome and ~90% of the maize *Copia* population, with the majority of copies accumulating during the last 600,000 years [24]. Moreover, experimental evidence suggests that Sireviruses are present in high numbers in other species such as legumes [20,31,32], beets [33], bananas [34], and agaves [35], while approximately half of the *Copia* sequences deposited in GenBank belong to this genus [26]. Therefore, through their widespread, intense and complex colonization patterns in plant genomes, Sireviruses seem to have been critical in the evolution of their hosts.

Sireviruses are also unique among LTR retrotransposons in terms of their own genome structure [23,25]. It is the only *Copia* genus whose members often possess a putative envelope-like (*ENV*-like) gene [36], which however shares little sequence similarity among elements apart from the presence of transmembrane and coiled-coil domains [19,27]. The origin of this *ENV*-like gene in Sireviruses (and some *Gypsy* plant LTR retrotransposons) is currently unknown. Moreover, apart from elucidating the mechanisms by which it is expressed (i.e. stop codon suppression, internal promoter) [28,37,38], its function (if any) has not been experimentally proven. As a result, its role remains highly controversial [39], and it is likely that it may not even represent a true envelope gene. Another intriguing characteristic of Sireviruses is the presence of a variety of highly conserved sequence motifs within their extremely divergent genome (Additional file 1: Figure S1), regardless of the evolutionary distance between their hosts. The motifs are located in key non-coding domains known to decisively participate in the life cycle of LTR retrotransposons, and may be the underlying factors for the affinity of Sireviruses for plants [23].

Due to their abundance and complex insertion patterns, efficient annotation of TEs is among the most cumbersome and problematic tasks of genome sequencing projects. Despite the use of several structural [40-42] and homology-based methods [43], identification is hampered or misguided by the often recombined, degraded and nested genome structure of LTR retrotransposons, or by their low copy number or uniqueness that renders them invincible to detection by comparison with previously characterized elements of abundant families. To alleviate such issues in the analysis of Sireviruses, we recently developed an algorithm able to identify Sirevirus elements with high accuracy and sensitivity [44]. Initially implemented in maize [24], it yielded >2,700 previously unidentified intact Sireviruses and offered insights into their crucial role in the evolution of the maize genome. Herein, the algorithm was updated and applied on a curated collection of 14 fully-sequenced plant and algal genomes to create MASiVEdb (Mapping and Analysis of SireVirus Elements Database).

MASiVEdb offers, through multiple and often novel ways, a comprehensive, highly curated and detailed report on the full-length Sirevirus complement of each species, while it additionally includes an integrated system for analyzing user-provided sequences against MASiVEdb elements. In this way, MASiVEdb is unlike any other TE database, thus complementing the efforts of research groups to collect and organize repetitive sequences. Such widely used and useful databases include the TIGR plant repeat database [45], Repbase [46], TREP (the Triticeae Repeat Sequence Database) [47], the maize TE database [48,49], the species-specific RetrOryza [50] and SoyTEdb [51] of rice LTR retrotransposons and soybean TEs respectively, and the GyDB *Gypsy* database [52].

This unique directory of Sireviruses can aid the scientific community in a variety of ways. Firstly, it is a consistent and up-to-date source of full-length Sireviruses (at present totaling 16,243 elements), which can significantly improve TE annotation not only of species currently included in the database but also of other plant genomes. Among other, the above will enable comparative TE studies at whole genome levels, and analyses of interactions between Sireviruses and host genes. Finally from an evolutionary perspective, MASiVEdb provides the foundation for studying the depth and impact of infiltration of this intriguing TE genus across plants, and for discerning what underlies their success or failure in massively colonizing different phylogenetic branches of the plant kingdom.

## Construction and content of MASiVEdb

Sequence data of twelve fully-sequenced plant genomes were downloaded from several websites (Table 1), alongside the genomes of two green algae representing the basal branch that gave rise to higher plants [53]. The algae were included as outgroups to investigate whether Sireviruses have been present during the time when land plants emerged.

**Table 1:** Properties of the host species and their Sirevirus populations included in MASiVEdb

**host species**^**a**^	**common name**	**clade**	**genome size (Mb)**^**b**^	**number of chr.**	**intact SVs**	**avg age (my)**	**with*ENV***
*Arabidopsis thaliana*	thale cress	eudicot	116	5	4	0.95	2
*Brachypodium distachyon*	brome	monocot	262	5	22	1.97	14
*Chlamydomonas reinhardtii*	n/a	alga	99	17	0	n/a	0
*Fragaria vesca*	strawberry	eudicot	203	7	1	0.45	0
*Glycine max*	soybean	eudicot	915	20	1337	0.45	1294
*Lotus japonicus*	lotus	eudicot	291	7	282	0.35	270
*Oryza sativa indica*	rice	monocot	362	12	25	2.18	14
*Oryza sativa japonica*	rice	monocot	370	12	91	1.29	42
*Ostreococcus lucimarinus*	n/a	alga	13	21	0	n/a	0
*Populus trichocarpa*	poplar	eudicot	304	19	0	n/a	0
*Sorghum bicolor*	sorghum	monocot	633	10	522	0.80	227
*Theobroma cocoa*	cacao	eudicot	214	10	77	3.17	52
*Vitis vinifera*	grapevine	eudicot	414	19	49	1.73	45
*Zea mays*	maize	monocot	1969	10	13833	1.29	516
**Total**					16243	1.20	2476

^a^The chromosome sequence data for *Arabidopsis* were downloaded from http://www.arabidopsis.org/, for brachypodium, *Chlamydomonas*, soybean, poplar, sorghum and grapevine from http://www.phytozome.net/, for strawberry from http://www.strawberrygenome.org/, for lotus from http://www.kazusa.or.jp/lotus/, for rice (*indica*) from http://rice.genomics.org.cn/rice/, for rice (*japonica*) from http://rgp.dna.affrc.go.jp/E/IRGSP/Build5/build5, for *Ostreococcus* from http://genome.jgi-psf.org/, for cacao from http://cocoagendb.cirad.fr/, and for maize from http://www.maizesequence.org/; ^b^The genome sizes do not represent the real estimates for each species, but correspond to the cumulative Mb found in sequence files for chromosomes after removing unanchored contigs and scaffolds; chr, chromosome; SVs, Sireviruses; my, million years; *ENV*, envelope-like gene.

All sequences were manually inspected to remove smaller contigs and scaffolds, properly formatted and split into chromosomes. For each chromosome, intact Sireviruses were identified and analysed with an updated version of the MASiVE algorithm [44]. MASiVE is based on the step-by-step identification of Sirevirus-specific and other critical sequence motifs of LTR retrotransposons, which have been shown to provide base-pair accuracy in outlining the element. The update mainly concerned the removal of a preliminary run of the LTRharvest algorithm [42] for detecting generic LTR retrotransposons. The exclusion of this step increased sensitivity without sacrificing accuracy, as was confirmed with large-scale manual inspection of the resulting data. Further improvements included optimized order of steps, element overlap detection, and data output. Specifically for the purposes of MASiVEdb we developed custom-built PERL scripts for the detection of the integrase (*INT*), monocot/eudicot *ENV*-like core domains, the multiple zf-CCHC motif of the *gag* gene [19], and the target site duplication of each element.

Intact Sireviruses were not detected in three out of the 14 species, the green algae *Chlamydomonas reinhardtii* and *Ostreococcus lucimarinus*, and the tree *Populus trichocarpa*, hence providing preliminary evidence that Sireviruses have neither been present when land plants emerged from algae, nor have they successfully colonized all branches of the plant kingdom.

The remaining 11 species provided a total of 16,243 full-length elements (Table 1), with a highly variable abundance, ranging from just one Sirevirus in strawberry and four in *Arabidopsis*, to 1,337 in soybean and 13,833 in maize. The total length of the detected elements exceeds 158 Mb. Sireviruses appear to have been active in different time periods in the genomes of their hosts, with an average insertion age of 0.35 million years ago (mya) in lotus (median of 0.16) to 3.17 mya in cacao (median of 3.06). There are also stark differences in the distribution of elements containing the *ENV*-like gene. Nearly all Sireviruses identified within the eudicot genomes of soybean, lotus, cacao and grapevine carry the *ENV*-like gene, in contrast to approximately half of the populations present in grasses, including brome, rice and sorghum. Notably, and as shown in recent work [24], the vast majority of maize Sireviruses are devoid of it. Given the availability of a large number of such sequences in the Sireviruses of MASiVEdb, as well as in *Gypsy* LTR retrotransposons and other TEs available in aforementioned databases, it may now be possible to elucidate its evolutionary origin and test recently formed hypotheses [54], and also investigate whether its function (if any) can attribute retrovirus-like properties to the carrier-elements.

All data were organized per species into tab-delimited, GFF-formatted, FASTA-formatted, and database-ready files. The latter were loaded into a two-table schema in the postgreSQL software system, whilst the rest are available for download as described below. Data are divided in four categories: ‘*basic*’, which includes the date of the run and version of the MASiVE algorithm used, the host species, Sirevirus identifier, chromosome, direction, coordinates, and distance to centromere (where available); ‘*advanced*’, which includes phylogenetic information on Sireviruses (currently unavailable – see Future development), presence of the *ENV*-like gene, age or time of insertion (in million years, e.g. 0.1 equals to an age of 100,000 years), and length of the element and its LTRs; ‘*genes*’, which includes the starting position (within the element) and length of the core domains of the reverse transcriptase (*RT*), *INT* and *ENV*-like genes; and ‘*motifs*’, which include the target site duplication, the primer binding site (PBS) sequence and starting position, and detailed analysis of the zf-CCHC motifs and multiple polypurine tract (PPT) signature [23]. The Sirevirus identifier of the MASiVEdb is constructed with the host species four- or five-letter code (also available online in the home page), and the direction and start coordinate of the element, e.g. Ljap_chr_3-D-12553650 stands for a lotus Sirevirus that was identified on position 12,553,650 of the sense strand (D for direct, in contrast to P for palindromic) of chromosome 3 of the lotus genome.

The GFF-formatted files, viewable in appropriate browsers, provide the coordinates, direction, identifier and length of the element and each LTR, plus the age of the element; and the coordinates, sequences, and direction of the motifs of the multiple PPT signature of each element. Finally, the FASTA-formatted files provide the sequences of the full-length element and its LTRs, the multiple PPT signature, the zf-CCHC motifs, and the core domains of the *RT*, *INT*, and *ENV*-like genes.

## Utility

**Figure 1:**
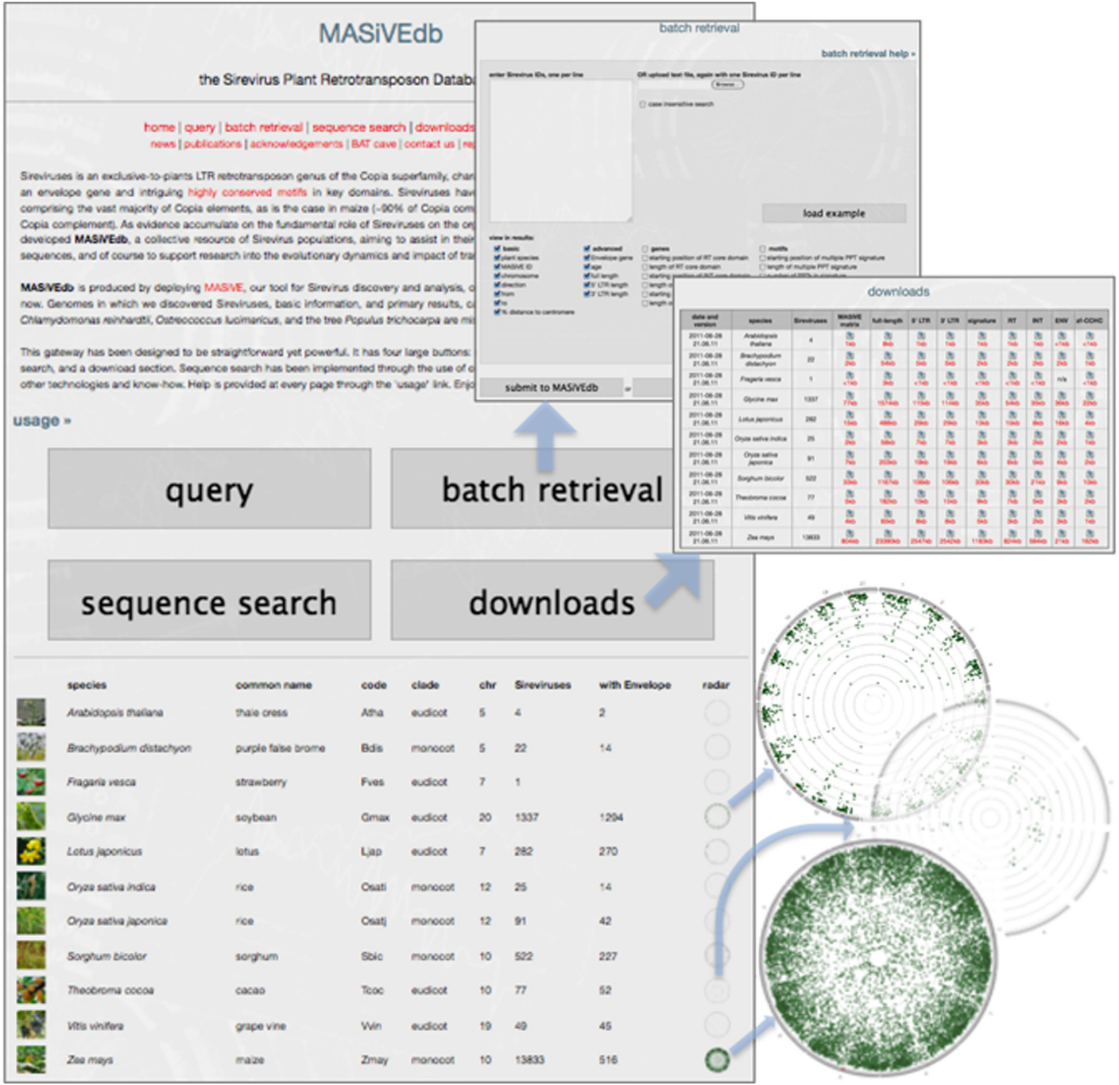
**The web interface of MASiVEdb.** The large buttons on the home page lead to the four main sections, including batch retrieval and downloads captured here. Clicking on the ‘radar’ icon of each species produces a Circos-based [55] image of the abundance, chromosomal localization and age distribution of its Sireviruses.

The top of the home page of MASiVEdb features a primary menu with the main sections, and a secondary menu with quick links to news, publications, acknowledgements (which include data sources, versions, dates and related information), and three pre-defined links for generic communication, error reporting, and genome inclusion requests (Figure 1). After a short introduction, four large buttons lead to the four main sections, presented below. Finally, a summary table provides information regarding the plant species and their Sirevirus content that are included in MASiVEdb, together with a Circos-based [55] circular representation of the Sirevirus abundance, localization and age across the chromosomes of each host.

### Query

**Figure 2:**
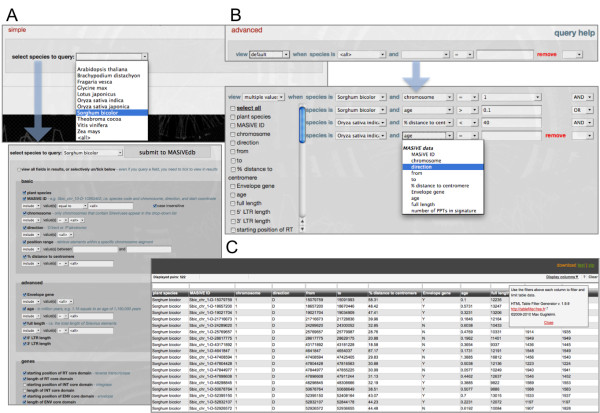
**The query interface and output of MASiVEdb.** (A) Simple form: the user has to select a host species or all species from the drop-down menu, which then opens an adapted list of choices and filters. (B) Advanced form: here users can retrieve multiple information from multiple species simultaneously. (C) The output matrix is common for both forms and permits further interactive processing (i.e. sorting and filtering).

MASiVEdb offers both a simple and an advanced query interface, providing access to the vast majority of information available. We advise users to familiarize themselves with the simple form, its content and structure, before attempting an advanced query. The simple form begins with the choice of species, either one or all (Figure 2A). This opens up a multitude of fields, divided in the four categories analyzed previously (i.e. *basic*, *advanced*, *genes*, *motifs*) that are either queryable by text or drop-down lists, or just selectable for viewing in results. Alternatively, the user can submit without input, in which case all Sireviruses for the selected species will be returned. The advanced form provides a more streamlined and powerful way of accessing the data (Figure 2B). Fields allow the selection of which data to show in results, from which species, and the constraints the user wants to impose based on different relationship and logical operators. Importantly, for assisting research in specific chromosome segments, there is an option in both forms for retrieving all data within a user defined sequence window.

The interactive output matrix contains the information requested by the user (Figure 2C), where additional filters can be used to further process the data. The results can be downloaded in tab-delimited text format, and the related sequences as FASTA-formatted files.

### Batch retrieval

The batch retrieval function (Figure 1), as the name suggests, allows the simultaneous querying of MASiVEdb with one or more Sirevirus identifiers, which means that these have to be available to the user through e.g. a previous query. Again, data to be returned in the results can be selected.

### Sequence search

We consider the sequence similarity-based access to the Sirevirus data among the most important aspects of MASiVEdb, for which we developed an integrated system collectively termed ‘LTRphyler’, and linked it to the database sequences. Through LTRphyler users can examine whether their sequences contain Sirevirus-related fragments, visualize the sequence similarity in a highly informative way, and infer the phylogenetic position of their query (if successful) within the *Copia* tree. More specifically, LTRphyler is a BLAST-based system that combines the use of Circoletto [56] for visualization, of the Wise2 package [57] for the detection of the *RT* and *INT* genes, and of MAFFT [58] for the construction of the *RT*- and *INT*-derived draft phylogenetic trees. The Circoletto visualization has been complemented with the highlighting of LTRs, the zf-CCHC motifs, and the *RTINT* and *ENV*-like core domains.

**Figure 3:**
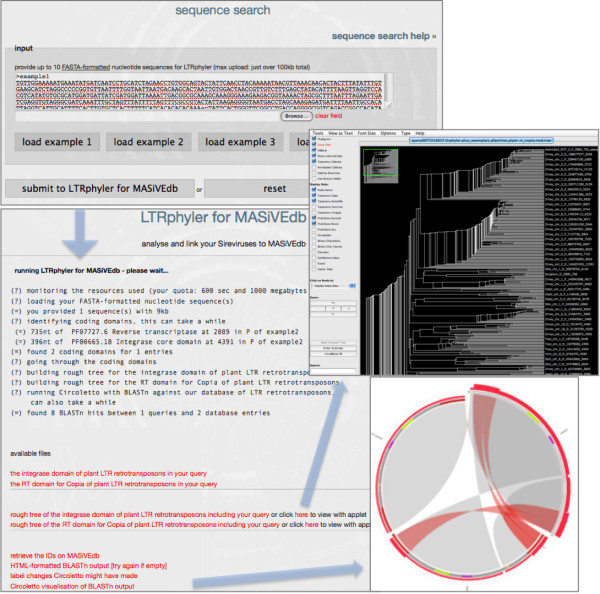
**The sequence search interface and output of MASiVEdb.** Besides reporting on the results of the analysis, the output page also provides links to the *RT*- and *INT*-based *Copia* phylogenetic trees and to the visualization of the sequence similarity through Circoletto.

Input are FASTA-formatted nucleotide sequences (Figure 3) assumed to contain intact or fragmented Sirevirus elements. The report includes information on i) the ‘health’ of the user sequences (i.e. if the input is in the wrong format, not a nucleotide sequence, or above the maximum length of 100 kb, then the program will report and exit), ii) the detection of the *RT* and *INT* core domains, with details of alignment length, direction, and coordinates, iii) the tree building for the *RT* and *INT* core domains detected in the query, together with >400 MASiVEdb exemplars, and a precompiled dataset of known Sireviruses and other *Copia* elements, iv) the run of Circoletto against MASiVEdb. Output might include: i) the FASTA-formatted sequences of the *RT* and *INT* genes in the query, ii) downloadable or applet viewable trees, iii) BLASTn output in HTML format, iv) a link to load the Sirevirus identities in ‘batch retrieval’, v) Circoletto visualization of the BLASTn results.

### Downloads

Finally, compressed data files (with file size) are available for download. They are divided per species and per content type, with information provided for the date of the run and the version of the MASiVE algorithm used (Figure 1).

## Discussion

Due to the difficulty in correctly assigning LTR retrotransposon families into the genera of the *Copia*/*Gypsy* superfamilies (which possibly contributed to the omission of a taxonomic step below ‘superfamily’ in the proposed TE classification system [22]), and to the scarce reference on the Sirevirus origin of elements, research on Sireviruses has been very limited so far. Hence, despite their abundance and wide distribution in plant genomes as suggested in a small number of earlier publications [26,27,32,33], the implications of their colonization dynamics are currently unknown. The recent discovery of their highly conserved genome structure [23,25], however, enabled their efficient and collective identification in one step [44], which has already proven crucial in elucidating their role in the structure and evolution of the maize genome [24]. Large-scale studies on other TE superfamilies like Helitrons [59-61] or subclasses like Pack-MULEs [62-64], which are distinguished by their structural characteristics or their amplification intricacies (i.e. carrying gene fragments), has shown that research at these higher classification levels can provide valuable insights into the mechanisms of plant genome evolution.

We argue that MASiVEdb is a step towards this direction. It represents the resource and methodological platform that can support research for uncovering the integrative impact of a specific TE genus on plant genomes - the first such attempt for LTR retrotransposons, excluding research on *Gypsy* chromoviruses [65,66]. In this respect, but also based on the functionality and technologies it incorporates, MASiVEdb is unique, and hence, complementary to the compendium of related databases [45-52]. Consequently, our aim for MASiVEdb is to assist other similar resources in untangling the complex genomic landscape of plants, by means of accurate annotation of TEs and genes, by assisting studies on their interactions, and by enabling whole genome comparative analysis of their TE complement. Such insights will gradually deepen as MASiVEdb will be continuously expanding its phylogenetic coverage (see below).

### Future development

We plan to periodically update MASiVEdb with Sireviruses from other plant genomes as they become available, so as to delve deeper into their distribution across plants, and possibly uncover more branches (like maize) where Sireviruses have aggressively amplified to achieve massive numbers, or others in which they have spectacularly failed to establish. We also intend to enrich the database with entries from various species where only limited sequence information is available.

The next major update of MASiVEdb will include a comprehensive phylogenetic analysis of its elements, and their categorization into families within and across species. Although such an analysis could have been performed relatively easily by either sequence clustering with a number of pre-annotated elements, or by construction of an e.g. *RT*-based tree, we strongly believe that more intricate sequence and genome characteristics of Sireviruses should be taken into consideration, a considerable undertaking out of the context of this first version of MASiVEdb. Finally, we expect in the near future to be able to incorporate sequence data of fragmented Sireviruses and solo LTRs.

## Conclusion

MASiVEdb is so far the most comprehensive directory for Sireviruses, an abundant and distinctive genus of plant LTR retrotransposons, in currently available fully-sequenced plant genomes. Although there are a number of databases (and methods behind them) dealing with the repetitive fraction of genomes, the methodology of MASiVEdb provides unprecedented accuracy in delineating and analyzing Sireviruses, in turn enabling robust and meaningful research into their own life and their impact on their hosts.

## Availability and requirements

MASiVEdb is freely accessible without any restriction to its use by non-academics at http://bat.infspire.org/databases/masivedb.

## Author’s contributions

AB conceived the study, collected and curated data, drafted the manuscript. EM designed and implemented the database and the web-accessible content, querying system, interfaces, and results presentation, and helped in drafting the manuscript. NK and MP assisted in data collection and algorithm development. AT curated data and assisted in algorithm development. ND coordinated the study, developed algorithms, and drafted the manuscript. All authors read and approved the final manuscript.

## Supplementary Material

Additional file 1 Phylogenetic and genome structure analyses within the *Copia* superfamily. This file contains the composite Additional file 1: Figure S1 that shows i) the phylogenetic relationships (based on the *RT* core domain) of exemplars from all three *Copia* genera, and ii) the highly conserved genome organization of Sireviruses and its comparison with the genome of other non-Sirevirus *Copia* elements (Figure adapted from [[24]]). Click here for file
