# Supplementary material for: MASiVEdb: the Sirevirus Plant Retrotransposon Database
Source: BMC Genomics. 2012 Apr 30;13:158. doi: 10.1186/1471-2164-13-158 (PMC3414828; doi:10.1186/1471-2164-13-158)
Supplement: Additional file 1 — Phylogenetic and genome structure analyses within the Copia superfamily. This file contains the composite Additional file 1: Figure S1 that shows i) the phylogenetic relationships (based on the RT core domain) of exemplars from all three Copia genera, and ii) the highly conserved genome organization of Sireviruses and its comparison with the genome of other non-Sirevirus Copia elements (Figure adapted from [[24]]). [file 1471-2164-13-158-S1.pdf]

A.

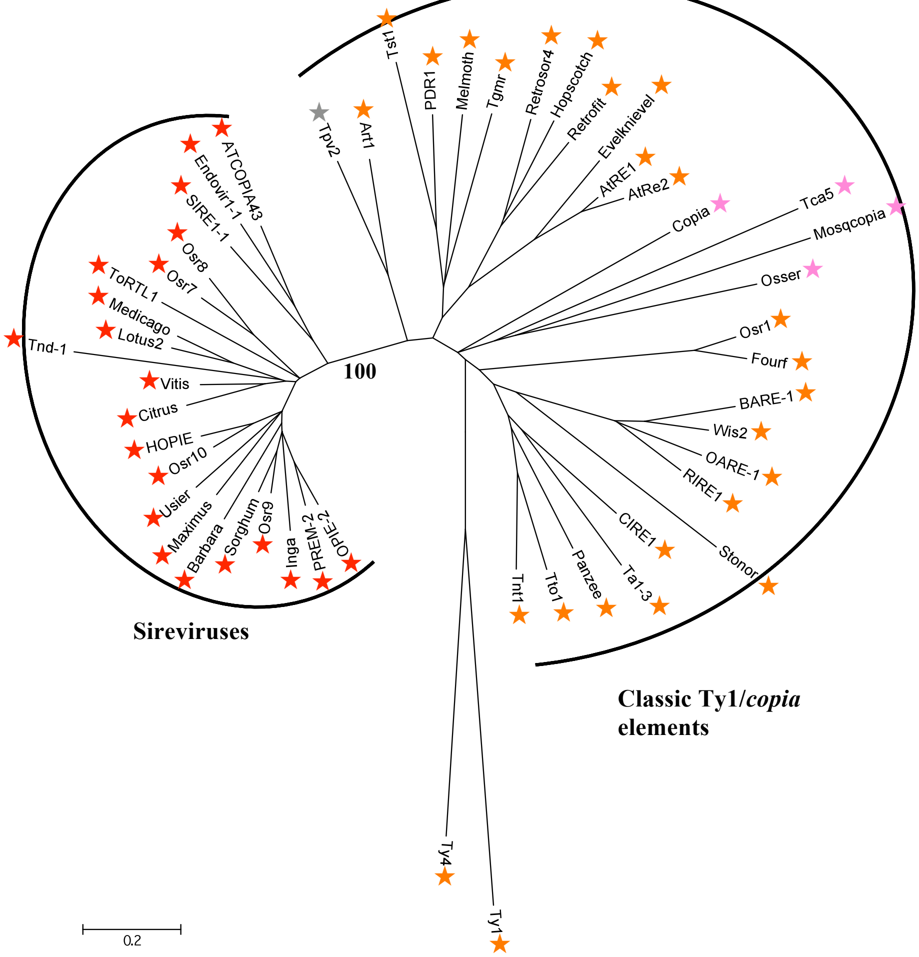

B. Genome organization of Sirevirus elements

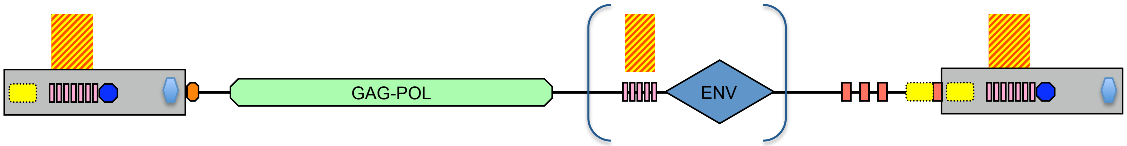

C. Genome organization of other members of the *Copia* superfamily

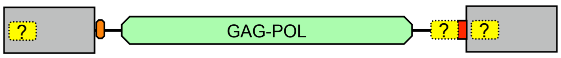

Figure S1. Phylogenetic and genome structure analyses within the *Copia* superfamily (Figure adapted from Bousios et al., 2012). (A) Exemplars from all three *Copia* genera, i.e. Sirevirus (red star), Hemivirus (pink star), Pseudovirus (orange star) and Tpv2 (grey star) that is of unknown classification according to ICTV (Boeke et al., 2006), were used for the construction of the *RT* phylogenetic tree. Sireviruses form a separate branch, which is supported with 100% confidence by the bootstrap analysis. There is no phylogenetic basis for separating the other two genera. (B, C) Genome organization of Sireviruses and other *Copia* elements. The *gag-pol* polyprotein is shown in green and the envelope-like gene (if present) as a light blue diamond. The inverted repeat (IR) arms (yellow) surround the internal domain/3' LTR junction of all Sireviruses and few *Copia* elements. The outmost 5' side of the junction is occupied by the terminal polypurine-tract octamer (PPT, in red), which precisely borders the IR left arm. The upstream PPTs cluster within the proximal 1000bp to the junction. The palindromic and putative *cis*-regulatory repeated motifs (RMs) (pink) are located within the first 200–700bp of the Sirevirus LTRs upstream of a highly conserved TATA box (blue circle), and at the 5' side of the envelope-like gene (when present). The RM clusters define the borders of CpG islands (orange bars). The 5' LTR/ internal domain junction harbors the conserved primer binding site (PBS, orange box), while the C-rich integrase signal (light blue hexagon) is located 20–30bp upstream of the 3' terminus of the Sirevirus LTRs. The genome size difference between Sireviruses (~10kb) and other *Copia* elements (~5kb) is approximately drawn to scale.
